# Intranasal Mucosal Boosting with an Adenovirus-Vectored Vaccine Markedly Enhances the Protection of BCG-Primed Guinea Pigs against Pulmonary Tuberculosis

**DOI:** 10.1371/journal.pone.0005856

**Published:** 2009-06-10

**Authors:** Zhou Xing, Christine T. McFarland, Jean-Michel Sallenave, Angelo Izzo, Jun Wang, David N. McMurray

**Affiliations:** 1 Centre for Gene Therapeutics, M.G DeGroote Institute for Infectious Disease Research, Department of Pathology and Molecular Medicine, McMaster University, Hamilton, Ontario, Canada; 2 Department of Microbial and Molecular Pathogenesis, Texas A & M University System Health Science Center, College Station, Texas, United States of America; 3 Institut Pasteur, Unité de Défense Innée et Inflammation et Inserm U874, Paris & Université Denis Diderot-Paris 7, Paris, France; 4 Department of Microbiology, Immunology and Pathology, Colorado State University, Fort Collins, Colorado, United States of America; 5 Department of Microbiology and Immunology, Dalhousie University, Halifax, Nova Scotia, Canada; Hannover School of Medicine, Germany

## Abstract

**Background:**

Recombinant adenovirus-vectored (Ad) tuberculosis (TB) vaccine platform has demonstrated great potential to be used either as a stand-alone or a boost vaccine in murine models. However, Ad TB vaccine remains to be evaluated in a more relevant and sensitive guinea pig model of pulmonary TB. Many vaccine candidates shown to be effective in murine models have subsequently failed to pass the test in guinea pig models.

**Methods and Findings:**

Specific pathogen-free guinea pigs were immunized with BCG, AdAg85A intranasally (i.n), AdAg85A intramuscularly (i.m), BCG boosted with AdAg85A i.n, BCG boosted with AdAg85A i.m, or treated only with saline. The animals were then infected by a low-dose aerosol of *M. tuberculosis* (*M.tb*). At the specified times, the animals were sacrificed and the levels of infection in the lung and spleen were assessed. In separate studies, the long-term disease outcome of infected animals was monitored until the termination of this study. Immunization with Ad vaccine alone had minimal beneficial effects. Immunization with BCG alone and BCG prime-Ad vaccine boost regimens significantly reduced the level of *M.tb* infection in the tissues to a similar extent. However, while BCG alone prolonged the survival of infected guinea pigs, the majority of BCG-immunized animals succumbed by 53 weeks post-*M.tb* challenge. In contrast, intranasal or intramuscular Ad vaccine boosting of BCG-primed animals markedly improved the survival rate with 60% of BCG/Ad i.n- and 40% of BCG/Ad i.m-immunized guinea pigs still surviving by 74 weeks post-aerosol challenge.

**Conclusions:**

Boosting, particularly via the intranasal mucosal route, with AdAg85A vaccine is able to significantly enhance the long-term survival of BCG-primed guinea pigs following pulmonary *M.tb* challenge. Our results thus support further evaluation of this viral-vectored TB vaccine in clinical trials.

## Introduction

Pulmonary tuberculosis (TB) has remained one of the leading infectious causes of death world-wide. BCG (Bacille Calmette-Guerin) has been used in human immunization programs as the only TB vaccine since 1921. However, while BCG is effective in protecting from severe or disseminated forms of childhood TB, it fails to effectively protect against adult pulmonary TB [1]–[3]. This may be due, in part, to the fact that BCG is administered in most countries shortly after birth but BCG-triggered protective T cell immunity diminishes markedly in 10–15 years. Thus, TB vaccine development efforts may need to focus more on the development of booster vaccines that can effectively enhance and extend immunity triggered by primary immunization with BCG or an improved BCG vaccine [4]. In this regard, it is now clear that BCG vaccine itself cannot be used as an effective boost vaccine [5], [6] and in fact, some experimental studies have indicated that BCG boosting of BCG-primed animals can be even deleterious [7], [8]. This suggests that effective booster vaccine candidates ought to be non-mycobacterial organism-based or heterologous to BCG in nature.

There are three major types of heterologous boost vaccine candidates: protein-, plasmid DNA- and viral-based vaccines. To adequately assess these vaccine candidates for their respective potential to be used for boosting purposes, a two-stage evaluation process is usually undertaken. First, they are evaluated as stand-alone vaccines for their relative immunogenicity and protective efficacy. Once the proof of principle is established, the selected candidate vaccines are tested in heterologous prime-boost protocols where BCG is used as a prime vaccine. The most commonly utilized animal species for such evaluations is the mouse due to its inexpensive nature and the availability of abundant immunologic reagents. However, compared to murine models, the guinea pig model is much more sensitive to pulmonary *M.tb* infection, thus representing a stringent animal model for further selection of promising vaccine candidates prior to their evaluation in humans [9].

We were the first to have developed a recombinant, replication-defective human type 5 adenovirus (Ad)-vectored TB vaccine expressing an immunodominant *M.tb* antigen Ag85A (AdAg85A) [10], [11]. We demonstrated that a single intranasal, but not intramuscular, vaccination with AdAg85A in a mouse model provided a level of protection against pulmonary *M.tb* challenge that was comparable to or even better than that by standard s.c BCG vaccination [10]. Such enhanced protection by mucosal immunization was mediated by persisting airway luminal T cells [12], [13]. We further demonstrated that intranasal mucosal boosting with AdAg85A enhanced protective immunity much more significantly than intramuscular boosting in BCG-primed mice [14]. While these murine data are encouraging, it remains unclear whether AdAg85A will be effective in guinea pig models. In our current study we have evaluated the efficacy of AdAg85A either as a primary or as a booster vaccine for BCG prime immunization in a stringent guinea pig model of pulmonary tuberculosis. We have also compared the relative efficacy by intranasal and intramuscular routes of immunization.

## Methods

### Animals

Outbred, specific pathogen-free guinea pigs of the Hartley strain (about 250–300 g) were purchased from Charles River Breeding Laboratories, Inc., Wilmington, MA, individually housed and maintained in a standard temperature, humidity and light-controlled environment. All of the procedures were approved by the Texas A&M University Institutional Animal Care and Use Committee.

### Vaccines and immunization regimens

A dose of 10^3^ cfu of Danish strain of BCG (BCG 1331) was injected i.d to each guinea pig for BCG immunization. A recombinant, replication-defective human type 5 adenovirus expressing *M.tb* Ag85A (AdAg85A) was developed and extensively evaluated in both murine and bovine models of pulmonary tuberculosis as previously described [10], [12]–[16]. A dose of 10^8^ pfu of AdAg85A was inoculated either i.m or i.n to naïve or BCG-primed guinea pigs. The choice of this dose was supported by pilot short-term survival studies where several doses of AdAg85A ranging from 10^5^ to 10^8^ pfu/guinea pig were tested and the 10^8^ pfu dose appeared to slightly better protect guinea pigs over the saline control or lower dose groups. Prior to inoculation, animals were lightly anesthetized i.m with a mixture of ketamine and xylazine (7.5 mg/kg and 0.625 mg/kg, respectively). For i.n delivery, 25 µl of diluted virus vaccine was administered to each nare for inhalational inoculation.

**Figure 1:**
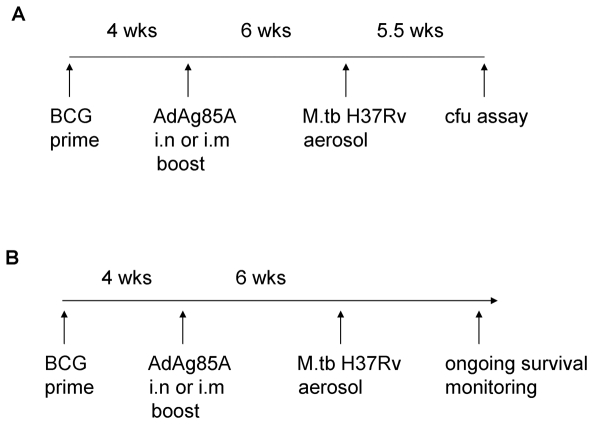
Experimental regimens used for both short-term and long-term guinea pig studies.

For short-term studies, groups of five guinea pigs were set up as naïve, BCG, AdAg85A i.n, AdAg85A i.m, BCG prime/AdAg85A i.n boost, and BCG prime/AdAg85A i.m boost. The booster was given at 4 weeks post-BCG priming. All guinea pigs were challenged with *M.tb* at 10 weeks from the point of initial immunization and euthanized humanely for cfu assay at 40 days post-*M.tb* challenge (Fig. 1A diagram). For long-term disease outcome studies, groups of eight guinea pigs were set up as exactly as for the above short-term studies except that the guinea pigs were not euthanized at 40 days post-*M.tb* challenge and were euthanized only when they reached the clinical disease endpoint or at the termination of study at 74 weeks post-challenge (Fig. 1B diagram).

### Pulmonary *M.tb* challenge

Both unimmunized and immunized guinea pigs were infected with a low dose (10^5^ cfu in the nebulizer to result in 10–15 cfu implanted to each animal) of virulent *M.tb* H37Rv (ATCC 27294) via the respiratory route by aerosol as previously described [17]. A Madison chamber (University of Wisconsin Engineering Shops, Madison, WI) was used for aerosol generation and calibrated for low dose delivery to the lung.

### Experimental endpoints

For short term studies, all guinea pigs were euthanized at 40 days post-*M.tb* challenge as described above. For long-term disease outcome studies, the animals were weighed prior to aerosol *M.tb* challenge and weighed weekly beginning at 10 weeks post-challenge. Determination of euthanasia was made by using a modified Karnovsky disease scoring method taking into account both significant body weight loss and the overall health condition of the animals including their respiratory rate, general behavior and physical appearance [17], [18]. Animals that demonstrated constant signs of disease progression were euthanized by i.p. injection with an overdose of sodium pentobarbital (Sleepway Euthanasia Solution, Fort Dodge, IA). Cardiac blood was removed using a heparinized syringe and sera were prepared and stored at −20 degrees.

### Tissue processing and bacterial colony formation assay

The lungs and spleens were removed aseptically. The right lower lobe of the lung and the 2/3's of the spleen were processed for mycobacterial colony formation assay as previously described [17], [18]. Briefly, tissues were homogenized in 4.5 mls of saline buffer and serial dilutions were plated in duplicate onto 7H10 Middlebrook agar plates and incubated in an incubator at 37°C for 21 days for cfu determination.

### Data analysis

The log rank analysis was carried out to determine the difference in survival between various treatments. Other data were analyzed and compared by using ANOVA and selected post hoc tests. The difference was considered statistically significant when p≤0.05.

## Results

### Effects of BCG priming and Ad boosting on tissue bacterial counts

**Figure 2:**
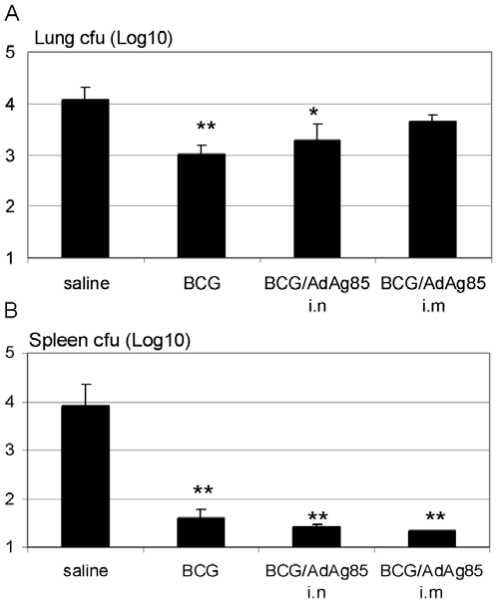
Levels of *M.tb* bacterial counts in the lung and spleen. The guinea pigs were treated as depicted in Fig. 1A and bacterial counts in the lung (A) and spleen (B) were determined at five and half weeks post-*M.tb* challenge by a colony formation assay. Results are expressed as means±SEM of five animals per group. **p≤0.01, *p≤0.05 compared to saline control.

To investigate the impact of various immunization regimens on the level of *M.tb* infection, guinea pigs were primed first i.d with BCG and subsequently boosted either intranasally (i.n) or intramuscularly (i.m) with AdAg85A vaccine (Fig. 1A). The control animals were treated only with saline (0.8%) or BCG. The animals were then challenged by *M.tb* aerosol at 6 weeks post-boosting. The level of mycobacterial infection was assessed both in the lung and spleen 40 days after challenge. Consistent with the previous reports, BCG alone significantly reduced bacterial burden both in the lung and spleen (Fig. 2A/B; p = 0.004 and p = 0.002 compared to saline control, respectively). In comparison, i.n or i.m with Ad vaccine alone did not reduce the level of infection in these tissues (data not shown). While immunization by BCG prime/Ad boost i.n or i.m only moderately reduced bacterial burden in the lung (Fig. 2A; p = 0.05 and p = 0.08 compared to saline, respectively), it markedly reduced bacterial counts in the spleen (p = 0.002 and p = 0.002 compared to saline, respectively), being even slightly better than BCG alone (Fig. 2B).

### Effects of BCG priming and Ad boosting on long-term disease outcome

**Figure 3:**
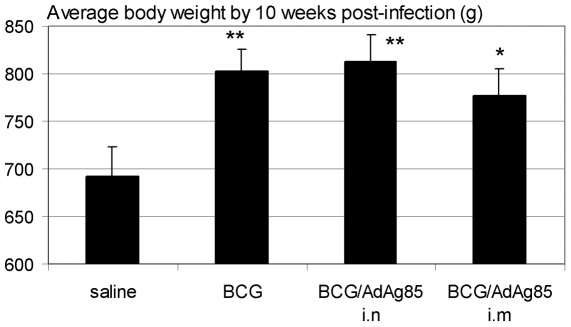
Comparison of average body weight changes at ten weeks post-*M.tb* challenge. Groups of guinea pigs were treated as depicted in Fig. 1B and body weight changes were monitored on a weekly basis. The data are the average body weight±SEM of each treatment group (eight animals per group) recorded at 10 weeks post-*M.tb* challenge prior to the occurrence of mortality in any treatment groups. **p≤0.01, *p≤0.05 compared to saline control.

As the bacterial loads in the tissue of guinea pig models may not adequately predict the protective efficacy of a test vaccine [19], in separate studies the long-term disease outcome was monitored following immunization and low dose *M.tb* aerosol challenge in a separate study. By the end of first 10 weeks post-infection, unimmunized guinea pigs already began to display deteriorating overall health conditions including much lower average body weight compared to the immunized groups (Fig. 3; BCG p = 0.008, BCG/Ad i.n p = 0.006 and BCG/Ad i.m p = 0.03 compared to saline control).

**Figure 4:**
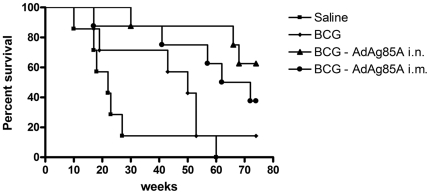
Kaplan-Meier curves of percent survival of *M.tb*-infected guinea pigs over the course of 74 weeks post-challenge. Groups of guinea pigs (eight animals per group) were treated as depicted in Fig. 1B and their survival rate was determined on a weekly basis up to 74 weeks when the entire study was terminated. p≤0.01 BCG, BCG/Ad i.n and BCG/Ad i.m compared to saline control; p≤0.05 BCG vs. BCG/Ad i.n; p≤0.1 BCG vs. BCG/Ad i.m.

**Figure 5:**
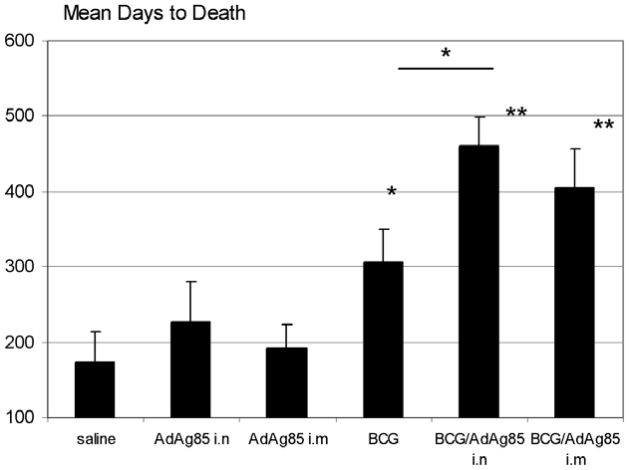
Comparison of Mean Days to Death of various treatment groups of guinea pigs. Mean Days to Death (MDD) was determined by dividing the summed total number of surviving days of each treatment group with the number of animals/group. Results are expressed as means±SEM of eight animals/group. Note: MDD for BCG/Ad groups may have been underestimated as 40–60% of animals in these treatment groups were still surviving at the time study termination (74 weeks). **p≤0.01, *p≤0.05 compared to saline control or between BCG and BCG/Ad i.n.

Between weeks 10 and 30 post-infection, the overall body conditions of unimmunized guinea pigs continued to deteriorate, resulting in greater than 80% mortality and, by week 60, all of the non-vaccinated guinea pigs succumbed (Fig. 4). Although the deterioration of health conditions of guinea pigs immunized i.n or i.m with AdAg85A vaccine alone somewhat lagged behind the saline group, the difference does not appear strikingly significant between these groups (data not shown; also see Fig. 5). In contrast, the majority of guinea pigs immunized with BCG, BCG/Ad i.n or BCG/Ad i.m remained healthy up to week 40 (Fig. 4; p = 0.0007 compared to saline control). However, by week 45, about 50% of BCG-immunized guinea pigs succumbed whereas the majority of BCG-vaccinated guinea pigs boosted i.n or i.m with AdAg85A vaccine remained healthy (Fig. 4). Throughout the course of study and until the point of study termination, the BCG-vaccinated guinea pigs boosted i.n with AdAg85A vaccine appeared to perform better than those boosted i.m with AdAg85A vaccine, although the difference was not statistically significant (Fig. 4). Between weeks 45 and 55, the majority of guinea pigs immunized with BCG alone also succumbed (Fig. 4). Thus, by the time when the study was terminated (74 weeks post-challenge), only about 10% of BCG-immunized guinea pigs were still alive. In contrast, more than 60% of BCG/Ad i.n- and 40% of BCG/Ad i.m-immunized animals were still living (Fig. 4; p = 0.03 BCG/Ad i.n compared to BCG alone; p = 0.1 BCG/Ad i.m compared to BCG alone).

### Effects of BCG priming and Ad boosting on Mean Days to Death

We further calculated the Mean Days to Death (MDD) for each treatment group. On average, unimmunized animals survived an average of 177 days whereas Ad i.n alone and Ad i.m alone only moderately enhanced the mean survival time to 229 and 192 days, respectively (Fig. 5). In comparison, BCG immunization alone increased the survival time to 308 days (p = 0.02 compared to saline group). AdAg85A vaccine i.n and i.m boosting to BCG-immunized animals further significantly enhanced it to 467 and 412 days, respectively although the differences between BCG and BCG/Ad i.m groups did not reach statistical significance (Fig. 5; p = 0.02 BCG/Ad i.n compared to BCG alone; p = 0.12 BCG/Ad i.m compared to BCG alone). It is noteworthy that the MDD for Ad vaccine boost groups are very likely underestimated since at the time of study termination, as a significant proportion of animals in Ad vaccine-boosted groups were still surviving (60% and 40% for BCG/Ad i.n and BCG/Ad i.m groups, respectively). Thus, clearly Ad vaccine boosting markedly improved the survival rate of BCG-primed guinea pigs.

## Discussion

The adenoviral vector is a promising vaccine platform for mucosal infectious diseases including pulmonary TB as it can be effectively administered via either the parenteral or mucosal route. [20], [21]. However, mounting evidence suggests that the mucosal route of vaccination is superior to parenteral vaccination in protecting mucosal surfaces [21]. Indeed, we have previously shown that a single respiratory mucosal dose of an adenovirus-vectored vaccine encoding an immune dominant mycobacterial antigen Ag85A (AdAg85A) provided robust protection against pulmonary tuberculosis which could be even better than BCG vaccination in a murine model of pulmonary tuberculosis [10]. Furthermore, we have demonstrated that respiratory mucosal delivery, but not intramuscular delivery, of AdAg85A remarkably enhanced protective immunity in BCG-primed mice [14]. However, whether this vaccine is effective in a more stringent guinea pig model has remained unclear. Investigating TB vaccines in guinea pig models of TB represents an important step in TB vaccine development as a vast majority of vaccine candidates shown to be effective in murine models have failed to pass the test in guinea pig models and are hence considered unsuitable for further evaluation in clinical trials [19]. This realization has prompted us to evaluate the efficacy of AdAg85A either as a primary or as a boost vaccine for BCG prime immunization in a guinea pig model of pulmonary tuberculosis. We have observed that while immunization with AdAg85A vaccine alone did not significantly improve the survival of infected guinea pigs, intranasal or intramuscular boosting with AdAg85A markedly improved the survival rate of BCG-primed animals over that by BCG immunization alone. Furthermore, intranasal boosting with AdAg85A appeared to be more robust than intramuscular boosting.

In contrast to our previous observations made in the mouse [10], [12], a single primary immunization with AdAg85A in guinea pigs, even via the intranasal route, failed to provide significantly enhanced protection. This is not entirely unexpected as guinea pigs are extremely susceptible to *M.tb* infection and many promising stand-alone candidate vaccines of genetic nature have failed to protect in guinea pig models [19]. As it is believed that BCG will continue to be used as a primary vaccine for human immunization and that effective boost vaccines need to be identified, our current finding that Ad vaccine-based boosting is effective to improve protection to BCG-primed guinea pigs, is significant. To date, only a very few candidate vaccines or vaccination strategies were found to be able to further improve the survival of infected guinea pigs over that by BCG immunization alone. These include a regimen of BCG prime and two repeated heterologous virus boost immunizations (MVAAg85A and FPAg85A) [19], [22], and co-injection of an adjuvanted polyprotein Mtb72F vaccine with BCG [23]. Based on these encouraging data from guinea pig models, both MVAAg85A and Mtb72F are under further evaluation in clinical trials [3], [24]. Of note, compared to MVAAg85A viral TB vaccine, our AdAg85A vaccine seems to be more potent in protecting infected guinea pigs as the former, when used as a standalone boost vaccine, failed to further enhance the survival of BCG-primed guinea pigs [19]. The robust boosting effect of AdAg85A delivery in prior BCG-primed animals is likely attributed to potent Ad-driven production of immunodominant *M.tb* antigen Ag85A and strong immune adjuvanticity of adenoviral backbone [11], [20], [21]. Indeed, the human type 5 adenovirus-based vector remains to be the most powerful gene transfer vehicle [20].

Previous studies have suggested that quantitative estimates of the bacterial burden in the tissues of guinea pigs may not be a reliable indicator of the protective efficacy of some TB vaccines, and that long-term disease outcome may be a more relevant readout of protective efficacy for such vaccines [19], [23], [25] Indeed, in our current study we observed very small differences in bacterial counts between BCG alone, BCG/Ad i.n boost and BCG/Ad i.m boost groups while BCG/Ad vaccine boost, particularly BCG/Ad i.n boost, markedly enhanced the survival of infected guinea pigs over that by BCG immunization alone.

Our previous study carried out in murine models has shown that intranasal, but not intramuscular, boosting with AdAg85A could dramatically further enhance protection in BCG-primed mice [14]. This represents yet another important difference between murine and guinea pig models as in our present study we have shown both intranasal and intramuscular boosting is effective in protracting the survival of BCG-primed guinea pigs although intranasal boosting appears to be more potent than intramuscular boosting (greater than 60% survival by i.n boosting vs 40% by i.m boosting at 74 weeks post-challenge). In fact, this finding is consistent with our recent cattle studies where parenteral AdAg85A boosting was found to be effective not only in enhancing memory T cell responses triggered by BCG priming [15] but also in enhancing protection from pulmonary tuberculosis (unpublished study). Together these findings from both guinea pig and bovine models raise the hope that parenteral boosting with virus-based TB vaccine will be sufficient to enhance protective immunity in previously BCG-vaccinated humans. Admittedly, the intervals between BCG priming and viral boosting in experimental animal models have been from one month to 3 months given practical considerations and a short life-span of animals, which cannot replicate what is expected to happen in human immunization program. In humans, we believe that an effective TB booster shall be given at least once at the time of adolescence since BCG-triggered protective immunity usually wanes 10–15 years after neonatal BCG vaccination [2].

Our current study represents the first to have compared side-by-side the effects of mucosal (intranasal) and parenteral (intramuscular) routes of booster immunization in the guinea pig. The fact that intranasal route of boost immunization with a recombinant virus-based TB vaccine appears to be more effective than the intramuscular route, further supports the general conclusion from our previous murine studies that respiratory mucosal vaccination is more effective than parenteral vaccination particularly when a genetic-based vaccine vector is used [2]. Our previous studies have demonstrated that intranasal delivery of recombinant adenoviral gene transfer vector primarily targets the epithelial cells of conducting airways and alveoli and to a lesser extent, alveolar macrophages [26], [27]. It is very likely that *M.tb* antigens secreted by infected epithelial cells and macrophages can be picked up by dendritic cells located within and beneath the epithelium. Infected macrophages may also turn into dendritic cells. The *M.tb* antigen-laiden dendritic cells thus can go on to activate T cells in the lung draining lymphoid tissues [28]. In murine models, the superiority of intranasal vaccination is attributed to the elicitation of long-lasting respiratory airway luminal antigen-specific T cells, particularly cytotoxic, IFN-γ-producing CD8 T cells [12], [13]. Due to the lack of immunological reagents for guinea pigs, we could not investigate whether this is also the case in intranasally AdAg85A-boosted guinea pigs. However, we speculate that a similar mechanism may be operating in these animals.

The last several years have seen a small number of promising candidate vaccines entering early phase of clinical trials [3], [24]. Among these vaccines are modified BCG vaccines, fusion protein vaccines and recombinant viral-vectored vaccines. A modified vaccinia Ankara viral vaccine (MVAAg85A) is the only candidate vaccine that has completed several phase I clinical studies and entered a phase II trial [29]. It is now widely accepted that it is imperative to have multiple promising TB vaccine candidates evaluated in early-phase human studies since in spite of the result from preclinical evaluation, the final verdict regarding the suitability of any vaccine candidate for mass human immunization program cannot be reached until it is evaluated in late phase, large-scale field trials. Thus, with the encouraging results from small to large-size animal studies, AdAg85A vaccine is about to be evaluated in a phase I clinical trial in Canada [30].
